# The Rise of the Consucrat

**DOI:** 10.34172/ijhpm.2020.36

**Published:** 2020-03-14

**Authors:** Evelyne de Leeuw

**Affiliations:** Centre for Health Equity, Training and Evaluation CHETRE, University of New South Wales, South Western Sydney Local Health District and Ingham Institute, Sydney, NSW, Australia.

**Keywords:** Consumer, Politics, Professionalisation, Representation, Medical-Industrial Complex

## Abstract

Some agents representing the ‘receiving end’ of the medical-industrial complex could be called ‘career consumers.’ We identify these consucrats as a new class of intersectional representation of ‘those affected’ in healthcare delivery systems. We describe them in the context of (similar) abocrats and femocrats but show that consucrats face more complex and different level intersectional challenges. The designation, professionalization, and representation of consucrats are problematic, in particular for public policy change. We argue for an enhanced strategic and cautious role for the consumer health movement to support consucrats.

## The Rhetoric of Consumer Representation

A strongly held belief that consumers need to be represented in health decision-making has become pervasive ever since the silent revolutions of the post-World War II era. This has led to the growth of what often is called the ‘health consumer movement.’^1,2^ The assumption – or belief – is that when the ‘consumers’ of particular (health or social) goods or services join forces, they can influence the delivery and quality of these for the better. They may even be able to influence public sector or organizational policy to generate systems that deliver the best outcome against the most affordable investment. This set of assumptions has been challenged, for instance by Löfgren et al^2^ who showed that these ‘consumer’ groups do not make better health policy. Rather, health consumer groups advocate for siloed, disease-specific and health specialty unique action. In fact, tangible, concrete and narrowly defined treatment and diagnostic options take prominence in the consumer health advocacy effort. The field is broad and fuzzy. Some representatives are clearly the embodiment of firm institutional interests, others only display deep personal commitment. Some project a distanced policy perspective, others demonstrate the physical and mental signs of ‘other-ness’ (eg, the disability and mental health communities^3^). Mostly these representatives are designated as individuals, although the literature increasingly demands community consumer voices.^4^ It appears that only occasionally systems and public policy views (eg, institutional design or finance parameters) are considered. In the process the health consumer organisation is exploited by commercial interests, as Batt^5^ demonstrates for the breast cancer industry.

The engagement, empowerment and representation of individuals and communities in health promotion continues to be ‘the holy grail.’^6^ True participation is still particularly urgent for the most disadvantaged in any society.^7^ A systematic review^8^ demonstrates increasing sophistication in the mechanisms and framing of the engagement of the *patient* (ie, an individual receiving diagnostics or treatment for ill-health). This work has been criticized to disregard the political and social determinants dimensions of engagement^9^: it claims that the framing of the problem precludes a strong focus on patient outcomes in a population health perspective. In short, an exceedingly clinical focus on a particular disease category stands in the way of efforts that would focus on a health policy that addresses the ‘upstream,’ ‘distal,’ political, social and commercial causes of the causes of ill health in populations. Consumer health advocates become complicit in extenuating the perverse effects of the medical-industrial complex.

What is perhaps most interesting in the mechanics of ‘consumer health’ is its institutionalization. In the Netherlands there is a ‘Patient Federation’ (with 200 institutional members from virtually any imaginable disease – or medical specialty – category). In Australia, the Consumers Health Forum of Australia is the national peak body to deliver consumer health representatives to organisations and elements in the healthcare system that – compelled by legislation or rhetoric – require ‘consumers’ on their governance rosters. The ‘consumer’ designation is not without problems, see Box 1.

**Box 1. Consumer, Client or Citizen?**

The terminology of the world of the consucrat is contentious. ‘Patient,’ it is clear, is deemed old-fashioned and passive.

Although ‘client’ and ‘consumer’ have attained mainstream status, they do resonate strongly with a capitalist and neo-liberal model of the relation between healthcare *delivery* and a *production* of health – clients and consumers are only one side of the medal, the other being suppliers and producers. This is a gross and possibly dangerous simplification of the realities of what makes and maintains health, and creates or sustains disease. This worldview rhetorically dismisses other players in the medical-industrial complex such as finance and insurance companies, Big Pharma and Big Tech, and a critical role for government.

To take health (and healthcare delivery) into account as a public good, and to embrace more deliberately the regulatory and possibly dampening role of government, some favour the term ‘citizen,’ highlighting their role and position as actors in the democratic and social process. Yet, there are many situations where large swathes of populations (eg, children, slum dwellers, homeless people, and in some cases people with particular – notably mental – disabilities) are *not* citizens and excluded from decision processes.

In a survey of preferred terms Lloyd et al^10^ also found ‘survivor,’ ‘mate,’ ‘person,’ ‘member’ and ‘friend’ as appropriate designations.

Finally, in a review of terminology in the (French) policy process literature Clavier and de Leeuw^11^ found that ‘those affected’ might be a value-free but potent term to describe the position and accountability of agents subject to the receiving end of policy intervention.

In many countries there is a codification of the importance of consumer and community involvement in healthcare delivery and research.^12^ ‘Consumer’ engagement in health service delivery is an important and critical element for reasons of transparency, accountability, quality assurance, equity, representation, and empowerment in the co-generation of thriving individuals, families, communities and societies. The peak bodies in Australia for medical research and consumer health advocacy frame benefits of consumer and community involvement^8^:

*Benefits to the public include:*

- *research being conducted that is relevant to community needs*
- *public awareness of, and support for, science and research, and*
- *more effective translation of research to deliver improved health outcomes.*

*Benefits to researchers and research institutions include:*

- *increased community relevance, through improved research priorities and projects informed by consumer and community perspectives and lived experiences*
- *public confidence in research through improved openness and transparency in the conduct of research*
- *public confidence in research through improved accountability and openness over the use of public money*
- *communities being better informed and having a greater understanding of research, and*
- *increased opportunities to continuously improve the quality of research.*

In this policy document, these ‘benefits’ are framed in a highly utilitarian, almost cynically market-oriented voice. The wider ranging societal benefits of a comprehensive representation of people in systems and institutions that otherwise may be perceived as elitist, detached, corrupted, and opaque are not represented here. Such a power-contextualised analysis also enables the identification of an issue with the semi-elitist proto professionalization of career consumer representatives in health systems as ‘invited’ by formal allopathic healthcare delivery systems.^13^ In Box 2 we present a vignette of one of many ways in which a ‘consumer’ is identified not by health interest representation, but by status networking potential, possibly reciprocally benefiting interests of both the healthcare institution and career stature of an individual. Let’s call these functionaries ‘consucrats.’

**Box 2. A Career Consumer**

**Vignette**

*
‘An exceptionally competent consumer’
*

**The scene:** Meeting of the Executive Board of a large multi-million dollar research partnership.

**The CEO:** “*I would like to introduce the Board to Baroness X who has kindly accepted our invitation to fill the consumer rep portfolio. She has a lived experience as a consumer in the mental healthcare system, and as you will all know, Mrs. X is extremely well-connected, and in fact was seen last weekend at a soiree with the First Lady, and a day later hosted a garden party herself where she mingled with brain surgeon Dr. Y and Oscar winner Z.”*

**The Board:** (polite applause).

## From Abocrat and Femocrat to Consucrat

There is a tradition to identify representatives of particular – marginal – communities within government bureaucracies as *crats. Analyses exist of what roles such *crats play, and how they are seen by their community peers outside the public administration machinery. The picture, generally, is bleak.

‘Femocrats’ are in more abundance than ‘abocrats’: in order to advance the cause of women in government policy many governments in the second half of the 20th century established units or departments called, eg, ‘The Bureau for the Advancement of Women.’

Typically, well-intentioned government agencies would seek to appoint women with a feminist agenda to these offices and services. The literature has designated these women as ‘femocrats.’^14^ Eisenstein^15^ views their role as ‘inside agitators’ but she also notes that they are mistrusted by hardline radical feminists. One could doubt whether femocrat appointments have led to gender mainstreaming in the public service.^16^ The femocrats in many ways are stateless: the traditional bureaucrats regarded them as ‘missionaries,’ whereas the women’s movement believed they had sold out to become ‘loyal mandarins.’^17^

Australia also witnessed the birth of the more derogatory ‘abocrat’ – an Aboriginal person appointed to represent Indigenous views in the usually white and racist^18^ government apparatus. The intersectionality of the experience is nevertheless analysed as potentially beneficial.^19^ Looking at a group of women Aboriginal bureaucrats the analysis finds ‘*The oppositional consciousness they developed by living in two worlds allowed the Abocrats to challenge fundamentally racist policies within the institutions in which they work*.’ Being an abocrat or femocrat is not an easy career pursuit.

The healthcare bureaucracy is an ecosystem with its own *crat: the consucrat. Consucrats are qualitatively different from abocrats and femocrats. The consumer representative is not an appointed and remunerated functionary, integrated into the healthcare bureaucracy. Rather they are considered a volunteer channel of the voice of the receiving ends of healthcare procedures and policies. Due to their embeddedness in the system they have grown to become co-opted apparatchiks who may rhetorically claim to speak truth to power, but may no longer be the representative voice of ‘the consumer.’

A further conceptual clarification is warranted. The *crat denomination suggests a home of the functionary in a classic government bureaucracy. This is particularly problematic in a post-modern 21st century context that recognizes the realities of network governance^20^ and intersectional identities. Stewart^13^ analysed different forms as tensions between citizen participation as (*a*) mutually agreed responsibility; (*b*) engagement in formal committee work; (*c*) commitment to community outreach activism; (*d*) a phenomenon of representative democracy; (*e*) an expression of protest; and/or (*f*) an effort at subversive service use. We will review these dimensions below along arguments around designation, professionalization, and representation. We will find that *crats typically transcend and exceed any public healthcare sector bureaucratic meme, and not just because healthcare itself is a convoluted mixture of public and commercial interests.

## Designation

Consumer consultation and engagement, even in the age of patient-centered models of care, still is more a gesture of benevolence on the part of the System than that it is a natural policy default.^21^ And as such the standards for the (self-)identification, (imposed or co-created) terms of reference and engagement parameters of the consucrat with both her/his charges (the medical-industrial complex on the one hand, and the community voice on the other) are fraught. As advocates for a particular treatment for a particular health threat or disease condition, individual consucrats may operate supremely well; they give voice to often passionate lived experiences. Löfgren et al^2^ thus identify very narrow agendas for disease cure campaigners (with the exception of Indigenist consumer analysts, who cast consumer health as a racist narrative, an Indigenous de-colonisation priority^22^).

It is hard to claim that consumer representatives are shaping public policy for health. They may influence procedural and operational *delivery system* parameters, but the way consucrats are identified, recruited and trained by the various intersecting systems they belong to does not suggest significant capabilities in the shaping and delivering of high level health promotion policy.

## Professionalisation

Engagement with individuals and institutions in the medical-industrial complex requires pertinent capabilities. As a system, medical care may have a tendency to medicalize rather than liberate peoples’ function and health potential.^23^ Medicalization, according to Conrad,^23^ happens at least at three levels: the conceptual, the institutional, and the interactional. In order to engage with the system as a good advocate and influencer, consucrats need to proto-professionalise. They will not de facto become doctors or nurses, but will need to master the (implicit) rules set for conceptual exchange, institutional establishment, and acceptable (symbolic) interaction.

Patient proto-professionalisation is described as a process in which laypersons learn to become experts in re-defining everyday troubles as problems amendable to treatment by a particular healthcare profession.^24^ Subsequently, the people who can re-frame their situation to the appropriate professional vocabulary have better access to care, are more likely to be found ‘suitable for treatment’ and thus benefit most of healthcare. Consucrat proto-professionalization will need to follow the same patterns for the functionary to become a reasonable and effective system counterpart. As with the femocrats and abocrats, the consucrats will need to manage the tense intersectionality that is involved: to what extent does one depart from the *language of the street* in order to engage with the *language of the system*? This seems the mirror task of Lipsky’s street-level bureaucrat^25^: where street-level bureaucrats interpret and amend formal policy to meet real need, healthcare consucrats push back against policy directives and operations that may not be in their interest. Consucrats may well sell out to the professional at the expense of the consumer…

Anecdotal evidence suggests that consumers in representative positions in the healthcare system ‘over-proto-professionalise’ and find themselves in challenging positions – not for the healthcare or public policy system, but rather for their own credibility as being grounded in concerns of those affected.

## Representation

What do consucrats represent, and how do they manage the complex interface between street-level worry and institutional arrangements?

Ideally, the consucrat is heard speaking on behalf of, and representing, a group or community that shares particular value systems and is legitimately concerned about being heard and respected by some higher abstraction of organisation (eg, *The Hospital*, *The Ministry*, or *The Agency*). In the same ideal world, the consucrat’s peers endeavour to analyse and frame their issues, wants and needs, and establish strategic and tactical ways of (re)constructing those. Such an approach would be beneficial to all; the organisation would receive significant and validated insights from the institutional representative, and the consucrats’ power of representation is enhanced and sustained through the support of their community.

However, the consucrat is as much exposed to challenges by The System to their autonomy and representation as Arnstein’s classic ‘Ladder of Participation’ suggests. In the consucrat’s interaction with The System and its professions it is problematic whether the consumer representative can be truly placed at the top rungs of the ladder – in *full control*, or *sharing systemic power* with the healthcare delivery organisation. At best, and as per declared intent and directive of consumer as well as healthcare peak bodies, the representative is to work *in partnership* toward consultation regarding the degrees of *information*, *therapy* and *manipulation* their patient colleagues receive in the healthcare delivery system.

The consucrat therefore finds her/himself in more ways intersectional than the abocrat or femocrat. Not only are they challenged in framing and positioning social and political identities, they are also constantly engaging at the intersection between the *operative* forms of participation. Implicit in power based emancipatory analyses of the role of patient and consumer advocacy is the notion of dialectic engagement or push-back. This means that in engaging with the system the push-back to manipulation might be counter-manipulation as well as partnership (Figure). Recalling Stewart’s work^13^ we maintain there is a potentially productive dialectic tension between what the (medical-industrial complex) System wants (ie, participation) and what the consucrat should deliver: countervailing power, protest, and alternate communications.

**Figure:**
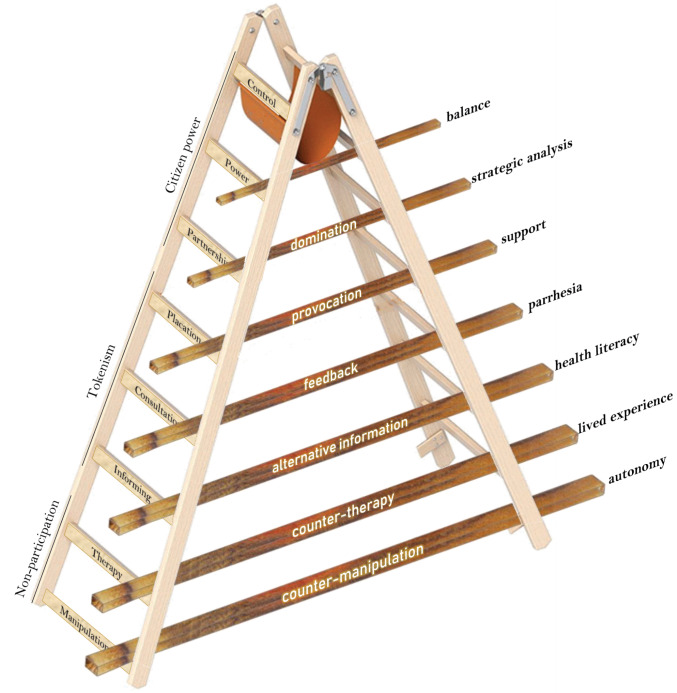
The Ladder of Participation^26^ as a Consucrat Challenge: Pushback Skills.

Thoughtfully crafted multi-dimensional strategic and operational intersectionality would require sustained systems support – not from healthcare, but from the organised and strong consumer institutional base. There seems to be little recognition of the challenges the individual consucrat may face. In a landmark publication, the *European Patients Forum* fails to identify issues of lip service, co-optation, misappropriated (proto-)professionalization, or biased policy advocacy, leave alone that it acknowledges potential insidious engagement of industry.^27^ Some authors blame such limitations and challenges purely on the complexity and diversity of the field^28^ without identifying the dialectic opportunity we suggested above.

It may well be that complexity and diversity are not the cause but rather the consequence of consucrat and institutional consumer health confusion.

## The Consucrat: What Is Next

Not all representatives of those affected by the healthcare system are fraught. Yet, the rise of the career consumer, here deemed ‘consucrat’ has been persistent. The acknowledgement of the critical importance of the voice of those affected (whether they are called stakeholders, consumers, patients, citizens, or go by any other term) has driven the recruitment and co-optation of consucrats into disease treatment and palliative systems. To optimize the efficiency of their inputs, deliberative processes, and preferred outputs and impacts, including public policy impact, they must become more astute at playing roles at several levels of intersectionality. Healthcare consucrats may struggle with the intersectional tensions between different (social, political, ethnic, and other) identities. Exceedingly, the (mostly unremunerated) consucrat must engage in complex juggling of procedural dimensions of healthcare delivery. We showed that the relatively straightforward hierarchical simplicity of Arnstein’s Ladder of Participation does not do justice to the complex – institutional and personal – demands put on the consucrat.

Consucrat peak bodies (eg, the *Health Consumers Forum of Australia* or the *European Patients Forum*) need to support their representatives at the pointy end of engagement in The System with better research and advocacy that responds to the intersectional challenge. Shaping policy is not just about advocacy. Shaping policy is about creating, monitoring and manipulating social and rhetorical networks for the purpose of exerting power and influence to pursue particular agendas. Individual consucrat talent or stature may be important, but are certainly not enough. In the unavoidable process of proto-professionalisation consucrats need support and a constant level of re-programming to maintain embeddedness in the activist policy agenda of the community they represent.

Unfortunately, the agendas of the individual consucrats and their organisations remain often too disease, healthcare delivery, diagnosis and treatment focused. This is understandable from an advocacy point of view as the input, throughput and output parameters of a health service delivery system seem more manageable than the messy realities of public policy development. However, in terms of ultimate community and population benefits as well as in terms of the longer term (rhetorical) sustainability of representative policy efforts a stronger social and political determinants of health perspective creates better foundations for overall health.^29^A social determinants of *health* (not disease) policy agenda will have a stabilizing and empowering effect on the connectedness and agenda-setting potential of both the individual as well as institutional consucrat. The consucracy and its institutions need to re-appraise their position in the deliberate policy engagement process.

## Ethical issues

Not applicable.

## Competing interests

Author declares that she has no competing interests.

## Author’s contribution

EdL is the single author of the paper.
